# Recommendations for a Brief International Cognitive Assessment for Multiple Sclerosis (BICAMS)

**DOI:** 10.1177/1352458511431076

**Published:** 2012-06

**Authors:** DW Langdon, MP Amato, J Boringa, B Brochet, F Foley, S Fredrikson, P Hämäläinen, H-P Hartung, L Krupp, IK Penner, AT Reder, RHB Benedict

**Affiliations:** 1 Royal Holloway, University of London, Surrey, UK.; 2 Careggi University Hospital, Florence, Italy.; 3 Meander Medisch Centrum, Amersfoort, The Netherlands.; 4 Universite de Bordeaux, France.; 5 Yeshiva University, Bronx, NY, USA and Holy Name Hospital Multiple Sclerosis Center, Teaneck, NJ, USA.; 6 Karolinska Institute, Huddinge University Hospital, Stockholm, Sweden.; 7 Masku Neurological Rehabilitation Centre, Masku, Finland.; 8 Heinrich-Heine-Universitat, Duesseldorf, Germany.; 9 Albert Einstein College of Medicine New York, USA.; 10 University of Basel, Switzerland.; 11 University of Chicago, USA.; 12 Jacobs Neurological Institute, New York, USA.

**Keywords:** multiple sclerosis, cognition information processing, memory, assessment, psychometrics, neuropsychology, SDMT, CVLT-II, BVMT-R

## Abstract

**Background:** Cognitive impairment in MS impacts negatively on many patients at all disease stages and in all subtypes. Full clinical cognitive assessment is expensive, requiring expert staff and special equipment. Test versions and normative data are not available for all languages and cultures.

**Objective:** To recommend a brief cognitive assessment for multiple sclerosis (MS) that is optimized for small centers, with one or few staff members, who may not have neuropsychological training and constructed to maximize international use.

**Methods:** An expert committee of twelve members representing the main cultural groups that have so far contributed considerable data about MS cognitive dysfunction was convened. Following exhaustive literature review, peer-reviewed articles were selected to cover a broad spectrum of cultures and scales that targeted cognitive domains vulnerable to MS. Each was rated by two committee members and candidates scales were rated on psychometric qualities (reliability, validity, and sensitivity), international application, ease of administration, feasibility in the specified context, and acceptability to patients.

**Results:** The committee recommended the Symbol Digit Modalities Test, if only 5 minutes was available, with the addition of the California Verbal Learning Test – Second Edition and the Brief Visuospatial Memory Test – Revised learning trials if a further 10 minutes could be allocated for testing.

**Conclusions:** A brief cognitive assessment for MS has been recommended. A validation protocol has been prepared for language groups and validation studies have commenced.

## Introduction and rationale

Cognitive impairment in multiple sclerosis reduces patients’ life satisfaction and health-related quality of life. It is probably the most important determinant of employment status and associated societal costs, and also adversely affects driving safety, household task completion, social activity, physical independence, rehabilitation progress, coping, treatment adherence and mental health.^1^ There is a high functional impact on young adults in demanding environments. Reported cognitive impairment prevalence rates are between 43% and 70%. They occur in all disease stages, including clinically isolated syndrome (CIS) and early relapsing–remitting MS (RRMS). Cognition is only loosely related to disease duration^2^ and physical disability (in some instances clearly dissociated)^3^ and is more strongly related to brain MRI parameters, especially atrophy.^4^

Cognitive deficits typically involve a few cognitive domains, spare language and are often undetected at consultation.^5^ Information processing speed is the most vulnerable cognitive ability, followed by episodic memory, and executive function.^6^ Deficits may be mild. There is high interpatient variability, in part due to varying compensation capacities (cognitive reserve).^7^ Patients may not be fully aware of their deficits, or may not report them reliably: depression results in over-reporting,^8^ whilst metamemory impairment and insight loss lead to underestimation in up to a third.^9^ MS cognitive deficits occur in the context of sensory and motor impairments^10^ and reduced functioning and engagement is easily attributable to physical disabilities. Cognitive impairment is not always at the forefront of the neurologist’s mind, although cognitive decline could be as important to the patient as physical relapses or MRI lesions.^11^

Addressing cognitive dysfunction is recognised as a quality indicator in MS care.^12^ However, psychometric assessment of cognitive status requires time-consuming expert evaluation with specialist materials. The most commonly utilised batteries of neuropsychological tests validated in MS are the 45-min Brief Repeatable Battery of Neuropsychological tests (BRB-N)^13^ and the 90-min Minimal Assessment of Cognitive Function in MS (MACFIMS).^10^ Comprehensive clinical cognitive assessment requires additional expertise in test selection, administration and interpretation. This is not routinely available outside specialist centres.^10^ Non-specialist cognitive evaluation tools are unsatisfactory. The Expanded Disability Status Scale offers only a rudimentary estimate of cognitive function.^14^ Widely used screening instruments, such as the Mini Mental State Examination, are insensitive to the MS cognitive footprint.^15^ All of the above considerations point to a clear need for a short, well-validated and widely accepted tool, which captures the cognitive performance of MS patients, and can be used in everyday practice by clinical neurologists or administered by local healthcare workers.^16^

## Objective

Our objective was to recommend a clinical tool for neurologists and healthcare professionals working with people with MS, which was not designed to be either a cognitive screen or full assessment, but rather a brief monitoring instrument. It would be optimised for centres where neuropsychologists are not available. Identifying a brief measurement tool with adequate reliability, validity, sensitivity and specificity would allow for more widespread, accurate cognitive evaluations. A validated record of cognitive disability incorporated into routine clinical practise would be beneficial. Baseline ratings and regular follow-up assessments would optimise patient management. Data from this cognitive monitoring tool could assist therapeutic decision making, including determining how best to support patients’ involvement in disease management. Information and counselling could be offered, facilitating adjustment at work and home. The effect of a start or shift in Disease Modifying Drug (DMD) treatment on cognition could be monitored, and also the use of cognition enhancers (provided that sufficient evidence of efficacy is achieved).^17^ The brief cognitive tool could also be integrated into more detailed, specialist cognitive assessments and used to indicate which patients require expert evaluation, targeting this resource more efficiently and equitably. From a global perspective, there are currently no cognitive measures for MS that are internationally validated and standardised, which is a challenge for international study design.

## Methods

A committee of seven neurologists and five neuropsychologists was convened, selected for their expertise in research and clinical aspects of MS cognition, and to represent the language groups who had so far contributed most significantly to the MS cognition literature. There were two co-chairs (one European, one American). A list of 80 scientific articles from peer review journals (http://msj.sagepub.com/content/early/recent) was assembled after a MEDLINE search in June 2010 by the co-chairs (D.L. and R.B.). These were chosen to represent a broad international spectrum of cognitive scales and their psychometric properties, including recent key review articles to allow the committee to identify any omissions. The full list was circulated to the committee and members were invited to suggest amendments and additions. Prior to the consensus meeting, a subset of articles was circulated to each committee member with standardised rating scales for completion. No member reviewed an article on which they were an author. Each article was rated independently by two committee members on three psychometric standards (reliability, validity and sensitivity) and four pragmatic standards (international applicability, ease of administration, feasibility in the specified context and acceptability to patients). All ratings were 3–1 (3 = excellent, 2 = satisfactory, 1 = unsatisfactory). The mean rating for each scale and each standard were calculated, and then the mean overall rating (MOR) for psychometric and pragmatic qualities separately. Raters were also invited to submit qualitative comments and judgements. Ratings and comments were collated for each candidate measure, and all collated ratings, from individual to mean scores, were presented to the committee and fully discussed.

### Consensus criteria

To meet the specified objectives of the cognitive monitoring tool, it was decided that the recommended battery should be completed in 15 min and not require any special equipment (beyond papers, pen and stop watch) or specific assessor training. The battery should be easily performed in a clinical setting. Discussions at the consensus meeting led to agreement that the domains of information processing speed, verbal memory and visual memory could be included. Executive function scales were felt to be too long and too challenging to administer in the target context. It was acknowledged that a wide variety of cognitive presentations can be seen clinically,^18^ but it was felt that a battery of scales addressing the three domains identified would capture a reasonable proportion of significant cognitive impairment in large clinical samples.

### Recommended monitoring tests

#### Information processing speed

There are two widely used tests of attention and processing speed in multiple sclerosis: the Paced Auditory Serial Addition Task (PASAT)^13^ and the Symbol Digit Modalities Test (SDMT, oral form).^19^ Both are included in the BRB-N and the MACFIMS. The SDMT achieved higher ratings, with a psychometric MOR of 2.8 and a pragmatic MOR of 3.0. In comparison, the PASAT achieved a psychometric MOR of 2.6 and a pragmatic MOR of 1.9. Discussion acknowledged that the SDMT is more congenial for both patient and assessor, takes less time to complete, requires less expertise and experience of the assessor and unlike the PASAT, does not require special equipment for auditory presentation of stimuli. It has equal psychometric validity to the PASAT.^20^ The committee considered the evidence that the PASAT has detected therapeutic efficacy of disease modifying medication on cognition,^21,22^ but felt that the SDMT was the better choice for the specified context on feasibility grounds.

**Figure 1.**
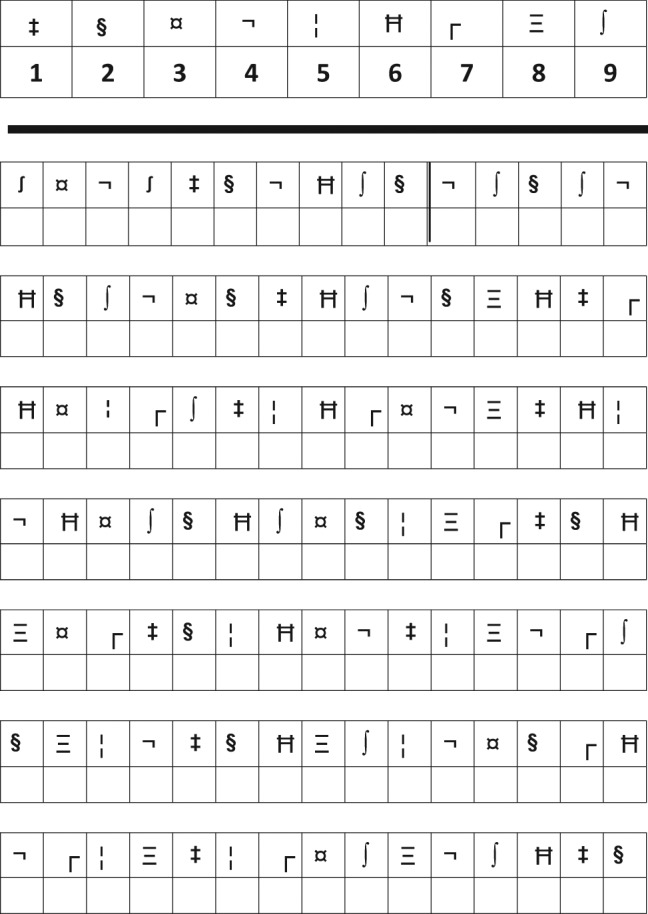
Example of stimuli of the SDMT type.

The SDMT^19^ was recommended as the test of information processing speed. The test consists of single digits paired with abstract symbols (Figure 1). Rows of the nine symbols are arranged pseudo-randomly. The patient must say the number that corresponds with each symbol. The SDMT can be completed within 5 min, including instructions, practice and testing. The good psychometric properties of the SDMT are well described.^23^ The SDMT has a reported sensitivity of 82% and a specificity of 60%.^24^ It has validations in several countries.^2,24–26^ Estimates of practice effects and change indices are available.^27^ The SDMT has a high sensitivity to cognitive impairment in MS.^6,26,28^ It has been shown to be the best predictor of MS cognitive impairment in both the BRB-N and MACFIMS.^6^ The SDMT is reliable when administered by nursing staff over several months, with minimal practice effects (0.2).^29^ There is also evidence for the sensitivity of the SDMT to cognitive change in MS.^2,25,30^ The SDMT is well validated against both conventional brain MRI parameters (including atrophy,^31^ brain parenchymal fraction (BPF) and third ventricular width,^32^ atrophy at baseline predicting SDMT change;^33^ cortical lesion number and white matter lesion volume,^34^ cortical lesion volume,^32,34.35^ cortical lesion volume change correlating with SDMT change,^36^ correlation with some deep grey matter (DGM) nuclei,^37^ including thalamic fraction;^32^ and fMRI (fractional anisotropy).^38^ The SDMT has also been shown to have external clinical validity, being significantly linked to both current^39^ and future^30^ employment status.

#### Verbal memory (immediate recall)

Again two candidate scales emerged: The California Verbal Learning Test-II (CVLT-II)^40^ and the Selective Reminding Test (SRT).^13^ The CVLT-II is in the MACFIMS and the SRT is in the BRB-N. The CVLT-II achieved a psychometric MOR of 2.9 and a pragmatic MOR of 2.6. In contrast, the SRT achieved a psychometric MOR of 2.5 and a pragmatic MOR of 2.2. It was noted that the SRT format required more expertise in administration and scoring, compared to the simple list recall format of the CVLT-II. The committee decided that the first five recall trials of the CVLT-II (CVLT-II T1-5) had sufficient psychometric rigour, in particular sensitivity to MS impairment,^41,42^ to be suitable for inclusion in BICAMS. CVLT-II T1-5 has been previously recommended as part of a brief MS cognitive assessment.^43^ The first five recall trials have a high degree of interdependence to other sections of the CVLT-II.^42^ Although this renders conclusions from the validity data based on the full CVLT-II inferential, it also reduces the range of cognitive processes involved.^41^ The recommendation carries the pragmatic advantage of reduced administration time (and possible patient fatigue effects), and a lesser requirement for assessor expertise and experience compared to the full CVLT-II, which suit our target context.

**Figure 2.**
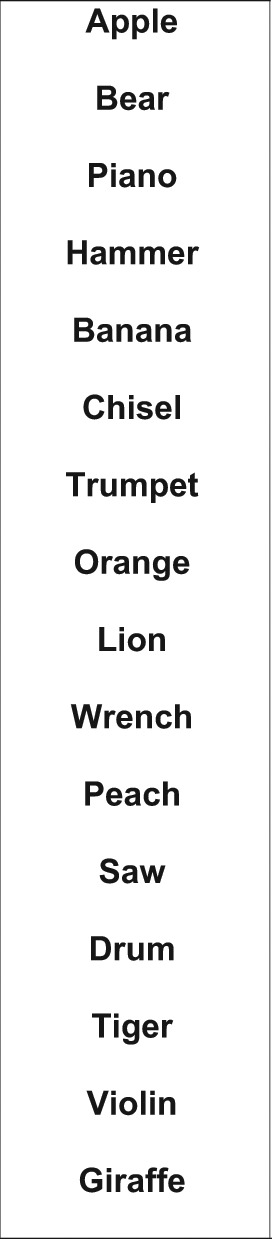
Example stimuli of the CVLT-II type.

The recommended verbal memory scale is the CVLT-II T1-5.^40^ This comprises a 16-item word list, with four items belonging to each of four categories, arranged randomly (Figure 2). The list is read aloud five times in the same order to the patient, at a slightly slower rate than one item per second. Patients are required to recall as many items as possible, in any order, after each reading of the list. The CVLT-II T1-5 can be completed in 5–10 min, including instructions, testing and responses. The CVLT-II T1-5 has been validated with brain MR total lesion area and right superior frontal atrophy,^44^ MR T1 and FLAIR lesion volume, BPF and third ventricular width^32^ and MR diffusion measures.^42^ The full CVLT-II has been validated against brain MRI parameters (cortical lesion number;^34^ correlation with some DGM nuclei volume,^37^ including thalamic fraction).^32^ The full CVLT-II also has external clinical validity, in differentiating employed MS patients from patients not employed due to MS.^41^

#### Visual Memory (immediate recall)

Once again, two major candidate scales emerged: the Brief Visuospatial Memory Test Revised (BVMT-R)^45^ and the 10/36 Spatial Recall Test.^13^ The BVMT-R is in the MACFIMS and the 10/36 is in the BRB-N. The BVMT-R achieved a psychometric MOR of 3.0 and a pragmatic MOR of 2.2. In comparison, the 10/36 achieved a psychometric MOR of 2.3 and a pragmatic MOR of 2.6. The committee’s discussion highlighted the reliability of the BVMT-R, the special equipment needed for the 10/36 and the possible ceiling effect on the 10/36.^7,28^ As in the deliberations about the verbal memory scale, the conclusion was reached that the first three recall trials of the BVMT-R (BVMTR T1-3) would be the scale recommended for BICAMS, with similar advantages and caveats to those stated above in connection with the CVLT-II T1-5.

**Figure 3.**
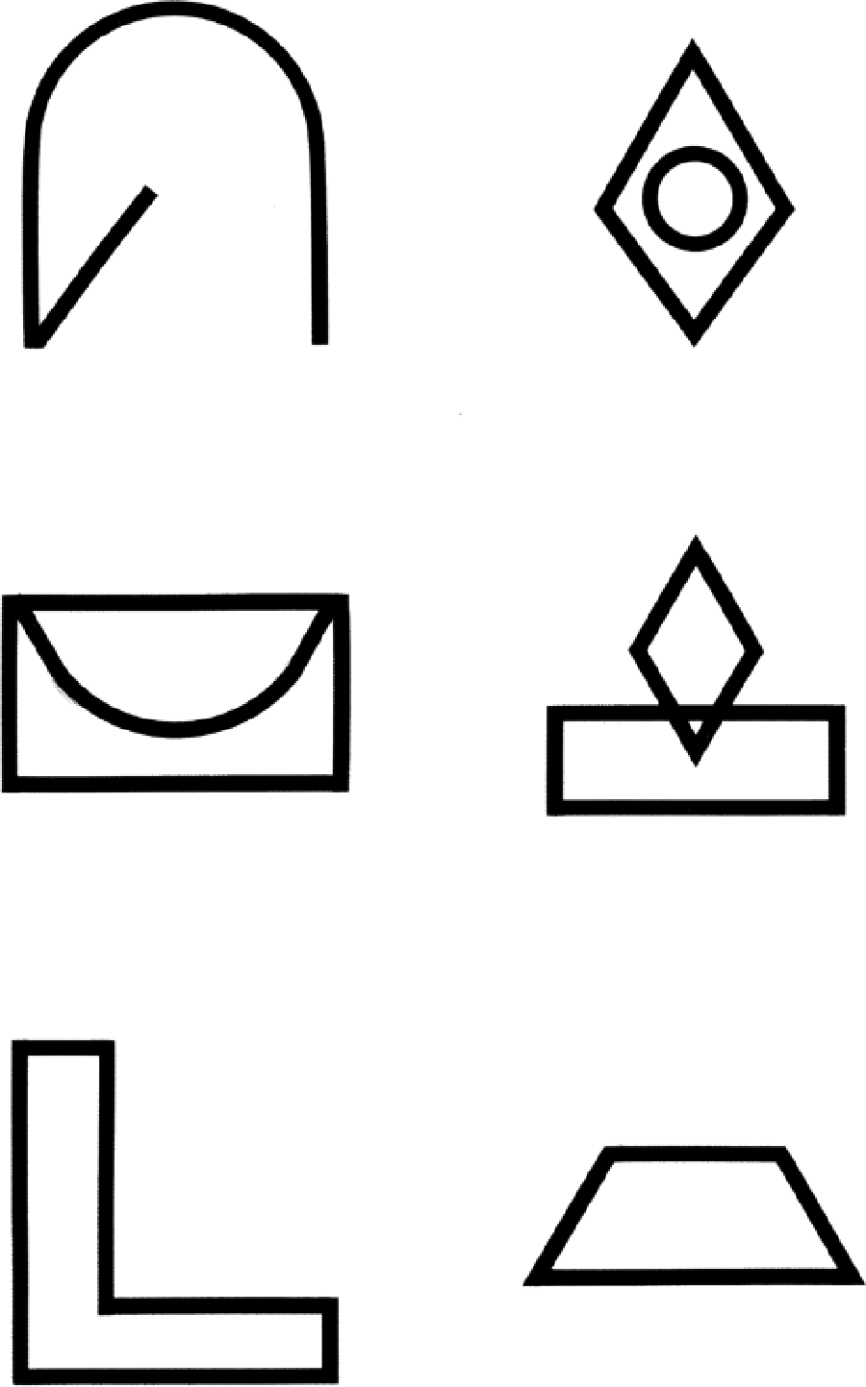
Example test stimuli of the BVMT-R type.

The BVMT-R T1-3^45^ is recommended. The BVMT-R T1-3 requires the patient to inspect a 2 × 3 stimulus array of abstract geometric figures (Figure 3). There are three learning trials of 10 s. The array is removed and the patient is required to draw the array from memory, with the correct shapes in the correct position. The psychometric properties of the BVMT-R T1-3 are good.^45^ Validity of the BVMT-R T1-3 has been indicated by significant association with brain MR total lesion area,^44^ T1 lesion and FLAIR lesion volume, BPF and third ventricular width^32^ and right superior frontal atrophy^44^ and correlation with some DGM nuclei,^37^ including thalamic fraction.^32^

## Implementation

For those health professionals with little experience of cognitive assessment, prior review of instructions and practice is recommended. Testing should take place in a quiet room, with just the patient and the assessor present. The purpose of BICAMS should be explained to the patient and if appropriate, some background information explaining cognitive difficulties in MS should have been made available to the patient in advance (e.g. www.stayingsmart.org.uk). It is recommended that BICAMS should not be used within 1 month of recovery from relapse (or if used, the data should not be interpreted as indicating long-term decline),^46^ or within 1 month of steroid therapy, which has a proven reversible detrimental effect on memory function.^47^ The recommended order of administration is first the SDMT, then if time allows the CVLT-II T1-5 and BVMT-R T1-3. In most clinical situations, yearly or bi-annual BICAMS evaluations will be appropriate.

### Confounds and limitations

BICAMS is not intended to replace a full neuropsychological assessment, which provides important additional insights to the patient, family, and clinician. These more detailed evaluations are necessary for in depth rehabilitation and vocational counselling and disability determination.

Neuropsychological test performance, including measures such as the SDMT, CVLT-II, and BVMT-R, is influenced by MS physical symptoms, demographic factors, the presence of concurrent neurological disorders other than MS, some concurrent medications, and to a modest degree depression.

#### Physical symptoms of MS

Whilst the BICAMS component scales have been selected to minimise the impact of physical impairments on cognitive test performance,^10,13^ the assessor should remain aware of confounds from MS symptoms. For example, dysarthria reduces performance on tests requiring a spoken response, especially if timed.^48^ Even mild MS visual impairments can also affect cognitive tests with visual stimuli.^49^ The experienced clinician can infer how a patient’s physical difficulties prejudice their test scores. Aside from the sensory and motor interface with the test situation, there are a number of physical symptoms of MS that can interfere with cognitive test performance, notably pain.^10^

#### Demographic factors

Demographic factors (age, education, and gender) affect cognitive performance. Many tests have normative data taking account of these variables.^50,51^ It is also well established that pre-morbid optimal level will affect how far current deterioration is detectable (cognitive reserve).^7^ Some countries have reading scales that estimate pre-morbid level.^10^ If testing materials are not available in the patient’s native language, particularly with respect to verbal learning and memory, published normative data cannot be used with any precision to evaluate the patient’s performance. The limited value of BICAMS for patients who are not native speakers is as a baseline against which to measure future change.

#### Concurrent neurological/medical disorders

If an MS patient has cognitive dysfunction, consideration must be give to whether other co-morbid neurological or medical conditions exist. In the context of learning disability, most mainstream intellectual assessments are inappropriate. It is helpful to ask whether or not a diagnosis of learning difficulty or attention deficit disorder was present prior to the MS diagnosis, or their characteristic features, since these conditions may have been present without formally diagnosis. Questions regarding difficulties in school performance or with reading can yield helpful information. The medical history should include questions regarding past head trauma, cerebrovascular disease, and other medical conditions such as sleep apnoea.

#### Concurrent medications

Certain medications commonly used in MS can adversely affect cognitive functioning.^52^ Benzodiazepines can interfere with vigilance and memory.^53^ For many patients high doses of anti-spasticity agents such as baclofen are associated with cognitive impairments.^54^ Anticonvulsants are also known to reduce cognitive function.^55^ Antidepressant medication can cause cognitive inefficiency to varying degrees.^52^ Cannabis, whether prescribed or self-medicated, can influence cognition.^56^ It is also important to be alert to other non-prescribed medication. Recent changes in cognition should be considered in the context of any concurrent medication changes.

#### Depression

Depression is highly prevalent in MS and may have mild effects on cognitive functioning, possibly mediated by coping styles.^57^ It is likely that severe depression could interfere with either willingness to undergo testing or ability to concentrate. An important aspect of depression’s influence on the clinical management of cognition, is that depression affects self-report of cognitive problems. Patients’ self-report of cognitive impairment does not reliably correlate with their performance at objective cognitive testing; in contrast, the relative’s report of the patient’s cognitive status does correlate with the patient’s performance at objective cognitive testing.^58^ Treatment of depression (and fatigue) improves patients’ self report of cognitive function, but not their objective cognitive test performance.^8^

#### Fatigue

Over 80% of MS patients experience fatigue and also have the impression that this symptom interferes with their cognitive functions.^59^ This association has not been consistently confirmed in studies of objective cognitive test performance. However, it is known that heat, infections, pain and depression can increase fatigue and these contributory factors should be managed to ensure optimum cognitive performance. When feasible, patients should be allowed to rest after arriving, before their cognitive assessment, to minimise the effects of fatigue.^60^ Those undergoing repeated testing should if possible be evaluated at the same time of day as the original assessment, to avoid variance from time of day fatigue effects.

## Future work and development of BICAMS

An international validation protocol for BICAMS is under development. Several national validation and standardisation projects are under way. It is envisaged that over time, many nations will be able to utilise BICAMS as part of routine clinical MS practice, referring to appropriate national norms. International MS natural history studies and treatment trials with cognition outcomes will be assisted by an internationally standardised battery. In order to support this process and facilitate access to materials and norms, the BICAMS committee is working to publish BICAMS updates in scientific and professional journals and meetings. The BICAMS website (www.BICAMS.net) will be a focus for this process, with a commitment to open access whenever possible.

## Summary

An expert consensus committee of neurologists and neuropsychologists, with extensive research and clinical experience of MS cognition, have recommended a Brief International Assessment of Cognition for MS (BICAMS).^16^ The battery takes 15 min to complete, requires no specialist equipment and no specialist expertise in cognitive assessment. BICAMS comprises:

- The Symbol Digit Modalities Test
- The California Verbal Learning Test –II, first five recall trials
- The Brief Visuospatial Memory Test –Revised, first three recall trials.
